# The molecular basis of spinocerebellar ataxia type 48 caused by a *de novo* mutation in the ubiquitin ligase CHIP

**DOI:** 10.1016/j.jbc.2022.101899

**Published:** 2022-04-07

**Authors:** A. Umano, K. Fang, Z. Qu, J.B. Scaglione, S. Altinok, C.J. Treadway, E.T. Wick, E. Paulakonis, C. Karunanayake, S. Chou, T.M. Bardakjian, P. Gonzalez-Alegre, R.C. Page, J.C. Schisler, N.G. Brown, D. Yan, K.M. Scaglione

**Affiliations:** 1 Department of Molecular Genetics and Microbiology, Duke University, Durham, North Carolina, USA; 2 Department of Pharmacology, University of North Carolina School of Medicine, Chapel Hill, North Carolina, USA; 3 Lineberger Comprehensive Cancer Center, University of North Carolina School of Medicine, Chapel Hill, North Carolina, USA; 4 Department of Chemistry and Biochemistry, Miami University, Oxford, Ohio, USA; 5 Department of Biochemistry, Medical College of Wisconsin, Milwaukee, Wisconsin, USA; 6 Department of Neurology, University of Pennsylvania, Philadelphia, Pennsylvania, USA; 7 Department of Neurology, Duke University, Durham, North Carolina, USA; 8 Duke Center for Neurodegeneration and Neurotherapeutics, Duke University, Durham, North Carolina, USA

**Keywords:** ataxia, E3 ubiquitin ligase, chaperone, neurodegeneration, neurodegenerative disease, CD, circular dichroism, CHIP, C terminus of Hsc70 interacting protein, FP, fluorescence polarization, SCA, spinocerebellar ataxia, SCA48, spinocerebellar ataxia type 48, SCAR16, spinocerebellar ataxia autosomal recessive type 16, *STUB1*, STIP1 homology and U box-containing 1, TPR, tetratricopeptide repeat

## Abstract

The spinocerebellar ataxias (SCAs) are a class of incurable diseases characterized by degeneration of the cerebellum that results in movement disorder. Recently, a new heritable form of SCA, spinocerebellar ataxia type 48 (SCA48), was attributed to dominant mutations in STIP1 homology and U box-containing 1 (*STUB1*); however, little is known about how these mutations cause SCA48. STUB1 encodes for the protein C terminus of Hsc70 interacting protein (CHIP), an E3 ubiquitin ligase. CHIP is known to regulate proteostasis by recruiting chaperones *via* a N-terminal tetratricopeptide repeat domain and recruiting E2 ubiquitin-conjugating enzymes *via* a C-terminal U-box domain. These interactions allow CHIP to mediate the ubiquitination of chaperone-bound, misfolded proteins to promote their degradation *via* the proteasome. Here we have identified a novel, *de novo* mutation in *STUB1* in a patient with SCA48 encoding for an A52G point mutation in the tetratricopeptide repeat domain of CHIP. Utilizing an array of biophysical, biochemical, and cellular assays, we demonstrate that the CHIP^A52G^ point mutant retains E3-ligase activity but has decreased affinity for chaperones. We further show that this mutant decreases cellular fitness in response to certain cellular stressors and induces neurodegeneration in a transgenic *Caenorhabditis elegans* model of SCA48. Together, our data identify the A52G mutant as a cause of SCA48 and provide molecular insight into how mutations in *STUB1* cause SCA48.

The spinocerebellar ataxias (SCAs) are a group of incurable, hereditary ataxias that cause degeneration of the brain. To date, 48 different SCAs have been identified with mutations in a wide array of genes. SCA48, the most recently described SCA, is an autosomal dominant disease caused by mutation of the STIP1 homology and U box-containing 1 (*STUB1*) gene (1, 2, 3, 4, 5, 6, 7, 8, 9, 10, 11, 12, 13, 14, 15, 16). In addition to SCA48, autosomal recessive mutations in *STUB1* also causes spinocerebellar ataxia autosomal recessive type 16 (SCAR16) (17, 18, 19, 20, 21, 22). While incidence of SCAR16 is very rare, recent work suggests that SCA48 may be a common cause of SCA (16). However, it is unclear how mutations in *STUB1* cause SCA48.

The *STUB1* gene encodes for the protein C terminus of Hsc70 interacting protein (CHIP). CHIP is an E3 ubiquitin ligase that sits at the interface of protein folding and protein degradation. CHIP recruits chaperones *via* a N-terminal tetratricopeptide repeat (TPR) domain and E2 ubiquitin–conjugating enzymes *via* a C-terminal U-box domain (23, 24, 25, 26, 27). Through the recruitment of these cofactors, CHIP facilitates ubiquitination of chaperone-bound client proteins resulting in their proteasomal degradation. CHIP-mediated degradation of chaperone clients plays a critical role in maintaining cellular proteostasis, and loss of CHIP function has been associated with decreased cellular fitness (28, 29, 30, 31, 32, 33, 34).

In addition to mutations in CHIP causing neurodegeneration, CHIP is also known to suppress neurodegeneration. In models of neurodegeneration, reducing CHIP levels accelerates disease phenotypes and results in increased protein aggregation and neurodegeneration (29, 30, 32, 35). Conversely increasing CHIP levels decreases protein aggregation and is neuroprotective (30, 36). Together, these data strongly argue that under normal conditions, CHIP plays a neuroprotective role.

Here, we have identified a *de novo* mutation in a patient with ataxia that results in a single point mutation (CHIP^A52G^). Using an array of biophysical, biochemical, and cell-based assays, we demonstrate that the CHIP^A52G^ mutant is defective in binding chaperones resulting in a defect in substrate ubiquitination. We also demonstrate that expression of the CHIP^A52G^ mutant results in decreased cell viability in response to certain cellular stressors. Finally, we have generated the initial animal model of SCA48 and demonstrated that this mutation results in neurodegeneration. Together, our results identify a novel mutation in STUB1 that causes SCA48 and provides mechanistic insight into how mutations in STUB1 cause SCA48.

## Results

### Identification of a *de novo* STUB1 mutation

**Figure 1:**
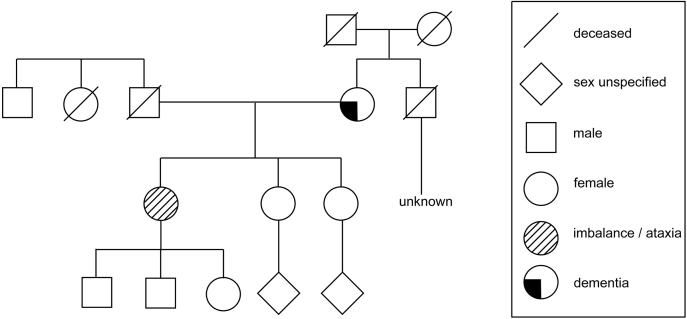
**Pedigree of the family.** The clinically affected individual is represented by *slashed lines*.

A 52-year-old female was diagnosed with mild dysarthria, mild gait impairment with gradual progression, difficulties with fine motor activities and hand dexterity, mild bilateral dysmetria on finger-chase, mild bilateral dysdiadochokinesia, and mild gait ataxia. An MRI of the brain revealed prominent pan-cerebellar atrophy (data not shown). There was no family history of ataxia, so clinical whole-genome sequencing was performed, and a heterozygous variant of unknown significance was observed in the *STUB1* gene encoding for a c.155C > G mutation that results in a single point mutation in the CHIP protein (A52G). Because dominant mutations in CHIP can cause SCA48, this suggests that the CHIP^A52G^ mutant may be a novel mutant that causes SCA48 (Fig. 1).

### The A52G mutation mildly destabilizes CHIP

**Figure 2:**
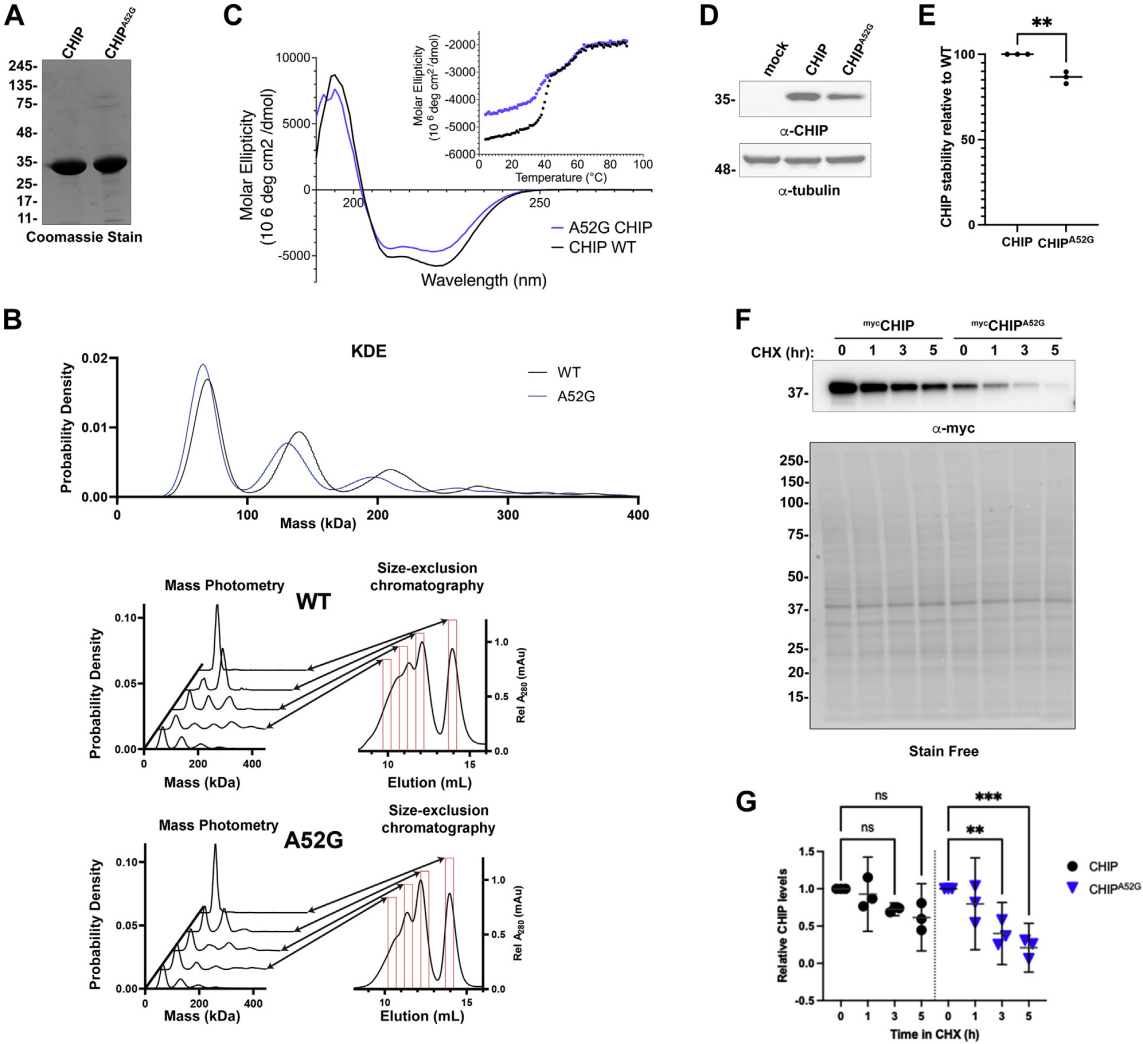
**The CHIP**^**A52G**^**mutant has slight decreases in helical content and is slightly less stable in cells.***A*, recombinant CHIP and CHIP^A52G^ were expressed and purified from *E. coli*. The mutant protein expresses well and is stable upon purification. *B*, the CHIP^A52G^ mutant oligomerizes in a manner similar to WT CHIP. Recombinant CHIP and CHIP^A52G^ were analyzed by size-exclusion chromatography, and fractions of CHIP present at different elution volumes were analyzed by mass photometry. No significant difference in CHIP^A52G^ oligomerization was observed. *C*, recombinant WT CHIP or CHIP^A52G^ were analyzed by CD to examine secondary structure. The CHIP^A52G^ mutant exhibits less helical content than WT CHIP and has similar stability with respect to secondary structure. Thermal stability of secondary structure elements for the CHIP^A52G^ mutant and WT CHIP were determined by thermal-melt CD analysis. *D*, the CHIP^A52G^ mutant has decreased steady-state levels in cells. HEK293 cells were transfected with either empty vector, CHIP, or CHIP^A52G^ for 48 h prior to collection of cells. Cell lysates were ran on SDS-PAGE and analyzed by Western blot with the antibodies indicated to determine the steady-state levels of both WT and mutant CHIP. *E*, quantification of E (n = 3; *p* = 0.0024), ∗∗*p* < 0.01. *F*, cycloheximide (CHX) chase assay of CHIP WT and A52G mutant. Cos-7 cells were transfected with indicated vectors. Twenty-four hours posttransfection, cells were treated with 50 μg/ml CHX for 0, 1, 3, and 5 h. *G*, densitometry analysis of three independent CHX chase assay experiments. CHIP protein levels were normalized with total protein loading as measured by Stain-Free gel imaging. CHIP, C terminus of Hsc70 interacting protein, ∗∗*p* < 0.01, ∗∗∗*p* < 0.001.

We previously found that most mutations in CHIP that cause SCAR16 result in aberrant oligomerization and destabilization of CHIP (37). To analyze the CHIP^A52G^ mutant, we first purified recombinant CHIP^A52G^ (Fig. 2*A*) and analyzed its oligomeric status by gel filtration coupled with mass photometry (Fig. 2*B*). Unlike most SCAR16 mutants, we found that the CHIP^A52G^ mutant did not have altered oligomeric properties (Fig. 2*B*). We next wanted to determine if the CHIP^A52G^ mutant had decreased secondary structure or thermal stability. To test this, we performed circular dichroism (CD) with thermal melt analysis. By CD, we observed less negative signal at 208 and 222 nm with the CHIP^A52G^ mutant compared to WT CHIP, consistent with a decrease in alpha helical content (Fig. 2*C*). Despite this decrease in alpha helical content with the CHIP^A52G^ mutant, we did not observe a significant difference in thermal stability between CHIP and CHIP^A52G^ with both proteins exhibiting three-state unfolding with transitions occurring at similar temperatures (Fig. 2*C* and Table 1).

**Table 1:** Melting temperatures of transitions 1 and 2 of CHIP and CHIP^A52G^

	1st transition	2nd transition
CHIP	42.0±0.5 °C	56.2±1.1 °С
CHIP^A52G^	41.5±0.7 °C	56.2±0.7 °C

To assess the CHIP^A52G^ mutant’s stability in cells, we transfected either WT or mutant CHIP into HEK293 cells and assessed their steady state levels by Western blot. In cells, we found that there was a very modest but significant decrease in CHIP^A52G^ levels when compared to WT CHIP (Fig. 2, *D* and *E*). Finally, to determine if this difference in levels was due to accelerated degradation, we next performed a cycloheximide chase assay and found that the CHIP^A52G^ mutant was degraded more rapidly in cells than WT CHIP (Fig. 2, *F* and *G*). Together, our data indicate there are no changes in the thermal stability or oligomeric status of the CHIP^A52G^ mutant, but there are mild defects in its secondary structure and stability in cells.

### CHIP^A52G^ has decreased affinity for chaperones

**Figure 3:**
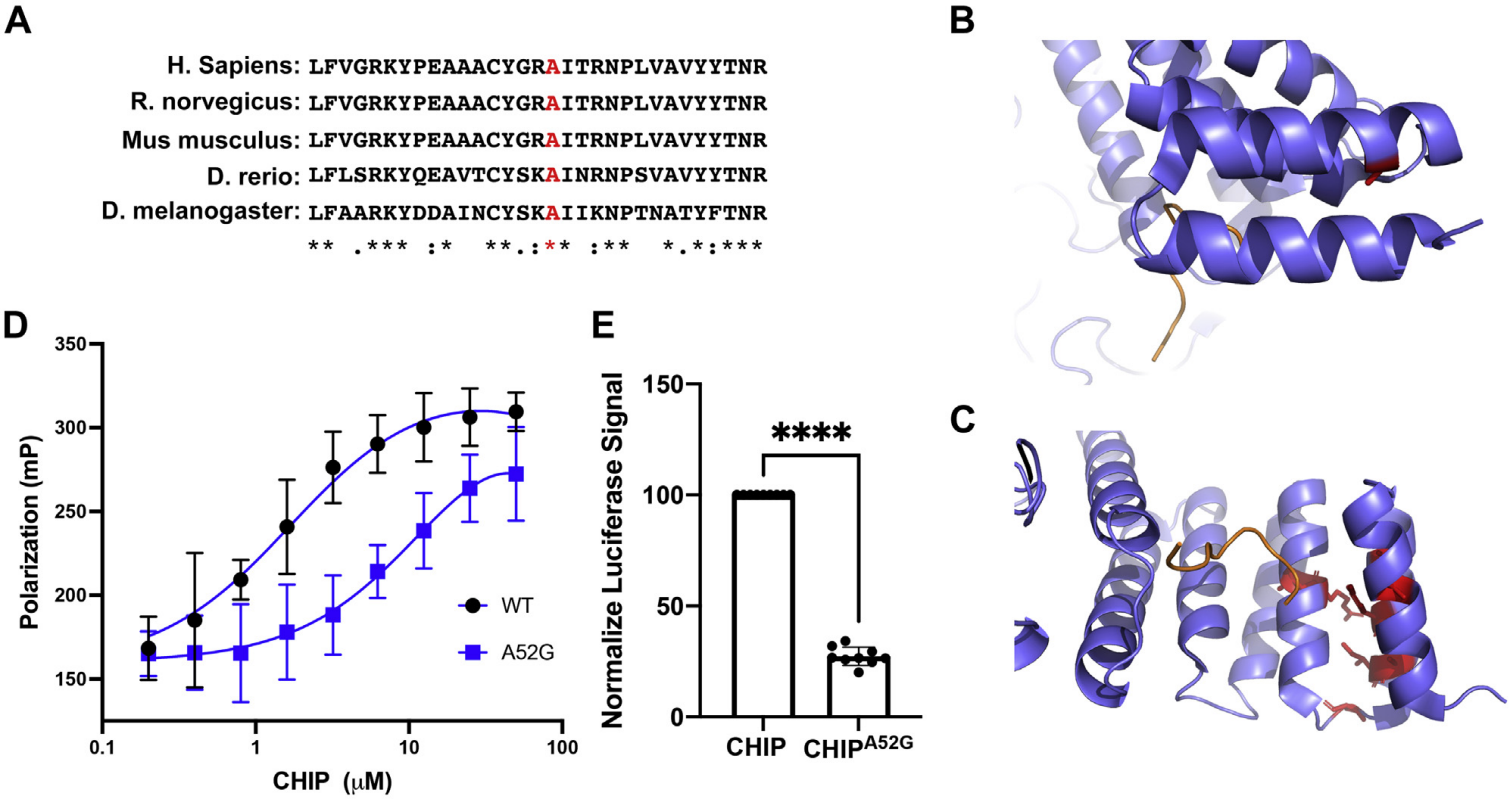
**The CHIP**^**A52G**^**mutant is defective in binding chaperones both *in vitro* and in cells.***A*, a sequence alignment of CHIP reveals that A52 is highly conserved. The protein sequence of CHIP from the indicated organisms was aligned utilizing Clustal Omega. *B*, residue A52 is buried in CHIP’s TPR domain. The structure of CHIP (pbd:2C2L) was analyzed, and residue A52 is shown in *red*. This residue is buried in the TPR domain. *C*, A52 clusters with other mutations that cause SCA48 and is expected to be important for the CHIP/chaperone interaction. The structure of CHIP (pbd:2C2L) was visualized bound to a C-terminal peptide of Hsp90 (*orange*). Residues that are mutated in SCA48 are shown in *red*. *D*, the CHIP^A52G^ mutant is defective in binding a peptide that mimics the C terminus of Hsc70 *in vitro*. Increasing concentrations of recombinant WT or mutant CHIP were incubated with a rhodamine-labeled peptide that mimics the C-terminal sequence of Hsc70. Samples were incubated at 37 °C for 30 min prior to being analyzed. The CHIP^A52G^ had an approximately 10-fold decrease in affinity for the peptide (n = 3). *E*, the CHIP^A52G^ mutant is deficient in binding Hsp70 in cells. The interaction between WT or mutant CHIP and Hsp70 was monitored in living cells utilizing the NanoLuc split luciferase system (Promega) (see Fig. S1). (n = 3; ∗∗∗∗*p* ≤ 0.0001). CHIP, C terminus of Hsc70 interacting protein; TPR, tetratricopeptide repeat.

We next wanted to determine what aspects of CHIP function were disrupted by the A52G mutation. Residue A52 is highly conserved and within the TPR domain of CHIP that is necessary for the CHIP/chaperone interaction (Fig. 3, *A*–*C*). This led us to hypothesize that this mutation would disrupt CHIP’s ability to bind chaperones. To test this, we utilized a fluorescence polarization (FP) assay to measure the affinity of CHIP for the C terminus of Hsc70. In this assay, we utilize a rhodamine-labeled peptide (r-SSGPTIEEVD) that mimics the C terminus of Hsc70. Importantly, this peptide encodes the EEVD motif that binds the TPR domain of CHIP and is responsible for most, but not all, of the affinity between Hsc70 and CHIP (38, 39). Similar to previous studies, we found that the peptide-bound CHIP with a K_d_ of 1.7 μM (Fig. 3*C*) (37). Consistent with the CHIP^A52G^ mutant having a defect in chaperone binding, the CHIP^A52G^ mutant had a roughly 10-fold decrease in affinity for the peptide with a K_d_ of 17.2 μM (Fig. 3*D*).

To confirm that the interaction between CHIP and Hsp70 was defective in cells, we utilized the NanoBiT split-luciferase platform to monitor the interaction between CHIP and Hsp70 in living cells (Fig. S1) (40). Consistent with our FP data, we found that CHIP^A52G^ had decreased luminescence compared to WT CHIP consistent with an impaired interaction with Hsp70 in cells (Fig. 3*E*). Together, these data are consistent with the CHIP^A52G^ mutation impairing the CHIP/chaperone interaction.

### CHIP^A52G^ retains ligase activity but has defects in substrate ubiquitination

**Figure 4:**
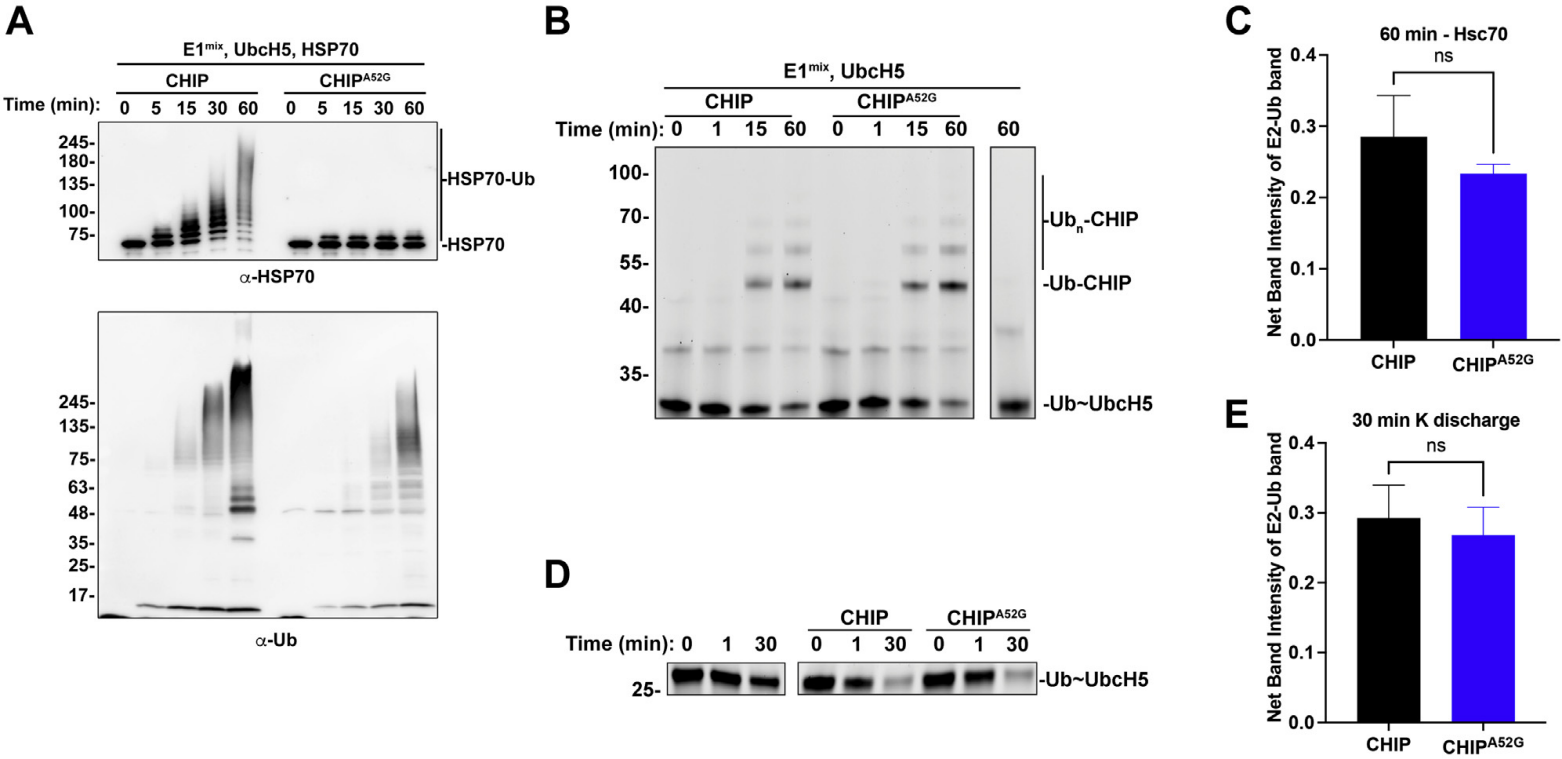
**The CHIP**^**A52G**^**mutant retains the ability to stimulate ubiquitin discharge but has defects in ubiquitinating substrate.***A*, the CHIP^A52G^ mutant has defects in ubiquitinating the CHIP substrate Hsp70. *In vitro* ubiquitination assays were performed for the time points indicated with either CHIP or CHIP^A52G^. Reactions were stopped by the addition of Laemmli buffer prior to analysis by SDS-PAGE and Western blot analysis with the indicated antibodies. While the CHIP^A52G^ mutant fails to efficiently ubiquitinate Hsp70, it does form ubiquitin chains as assessed by probing with an antiubiquitin antibody (n = 3). *B*, the CHIP^A52G^ mutant has no defect in autoubiquitination. Ubiquitination reactions were performed in the absence of substrate with fluorescein-tagged ubiquitin and autoubiquitination of CHIP was monitored by fluorometry. No defects in autoubiquitination were observed. *C*, quantification of B. *D*, the CHIP^A52G^ mutant stimulates discharge of ubiquitin from UbcH5 in the presence of free lysine. Lysine discharge assays were performed in either the absence of CHIP (*left*) or in the presence of either CHIP (*middle*) or CHIP^A52G^ (*right*). Both CHIP and CHIP^A52G^ stimulate the discharge of ubiquitin from UbcH5 in the presence of free lysine. *E*, quantification of D. CHIP, C terminus of Hsc70 interacting protein.

CHIP binds chaperones and ubiquitinates their client proteins targeting them to the proteasome for degradation. We next wanted to determine if the CHIP^A52G^ mutant retained ubiquitin ligase activity. To accomplish this, we performed *in vitro* ubiquitination assays using either CHIP or CHIP^A52G^ and analyzed ubiquitination of Hsp70, a bona fide CHIP substrate, by Western blot (33). While CHIP readily ubiquitinated Hsp70, the CHIP^A52G^ mutant was largely deficient in ubiquitination of Hsp70 (Fig. 4*A*). Because we previously observed that the CHIP^A52G^ mutant was deficient in binding chaperone proteins, we wanted to determine if the CHIP^A52G^ mutant was truly deficient in ubiquitin transfer or if the reason we observed deficient Hsp70 ubiquitination was due to Hsp70 not efficiently binding CHIP^A52G^. To accomplish this, we probed our ubiquitination assay for ubiquitin. Consistent with the CHIP^A52G^ mutant retaining ubiquitin ligase function, we observed that the CHIP^A52G^ mutant did stimulate ubiquitin chain formation; however, less signal was observed with CHIP^A52G^ when compared to CHIP (Fig. 4*A*).

This decrease in signal could be due to either decreased ubiquitin ligase activity or it could be due to CHIP more efficiently ubiquitinating Hsp70 than autoubiquitinating itself or forming free ubiquitin chains. To differentiate between these possibilities, we next performed ubiquitination assays in the absence of Hsp70 using a fluorescently labeled ubiquitin and compared CHIP and CHIP^A52G^ autoubiquitination. In the absence of Hsp70, we observed that there was no difference in CHIP and CHIP^A52G^ autoubiquitination consistent with the CHIP^A52G^ mutant retaining E3 ubiquitin ligase activity (Fig. 4, *B* and *C*). To confirm that the CHIP^A52G^ mutant retained full E3 ligase activity, we next performed lysine discharge assays to measure the ability of CHIP and CHIP^A52G^ to stimulate discharge of ubiquitin from the active site of the E2 ubiquitin-conjugating enzyme. In this assay, CHIP and CHIP^A52G^ both efficiently stimulated the discharge of ubiquitin in the presence of lysine (Fig. 4, *D* and *E*). Together, these data indicate that the CHIP^A52G^ mutant retains activity as a ubiquitin ligase but is deficient in recruiting chaperones and therefore loses the ability to ubiquitinate chaperone clients.

### CHIP^A52G^ expression decreases cell viability with a subset of cellular stressors

**Figure 5:**
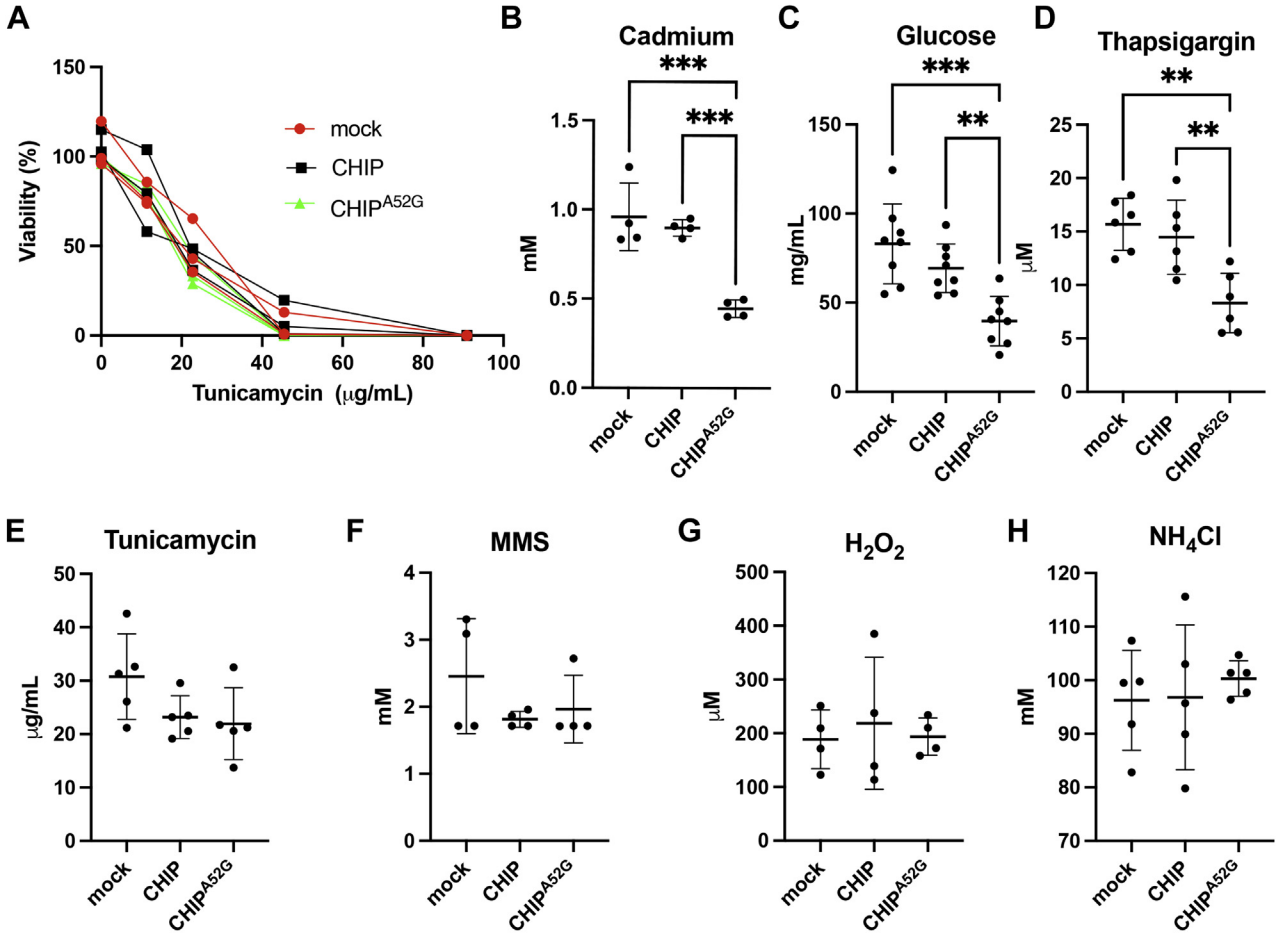
**The CHIP**^**A52G**^**mutant decreases cell viability under certain conditions of cellular stress.***A*, a representative cell-survival curve at various tunicamycin concentrations with HEK293 cells expressing either empty vector, CHIP or CHIP^A52G^. *B–H*, cells were treated with various stressors including cadmium (*B*), glucose (*C*), thapsigargin (*D*), tunicamycin (*E*), MMS (*F*), H_2_O_2_ (*G*), NH_4_Cl (*H*) prior to analysis of cell viability using Cell Titer Glo (Promega). In the presence of the CHIP^A52G^ mutant, cells stressed with cadmium, glucose, or thapsigargin experience increased cell death. CHIP, C terminus of Hsc70 interacting protein; MMS, methyl methanesulfonate, ∗∗*p* < 0.01, ∗∗∗*p* < 0.001.

Because CHIP plays a critical role in protecting cells from cellular stress, we next wanted to further test this and determine if the CHIP^A52G^ mutant decreased cellular fitness. To accomplish this, we stressed cells with a panel of stressors and measured the LD_50_ for each stressor (Fig. 5, *A*–*H*) (41). Consistent with the CHIP^A52G^ mutant having a toxic gain of function or a dominant negative effect, we found that expression of CHIP^A52G^ decreased cellular fitness when cells were challenged with a subset of stressors including high levels of glucose, thapsigargin, and cadmium (Fig. 5, *B–D*). Of note, expression of CHIP^A52G^ did not decrease cellular fitness to all stressors as no difference in LD_50_ was observed when cells were treated with tunicamycin, methyl methanesulfonate, hydrogen peroxide, and ammonium chloride (Fig. 5, *E–H*). Together, these data suggest that the CHIP^A52G^ mutant has either a toxic gain of function or a dominant negative effect and decreases cellular viability under certain conditions of cellular stress.

### The CHIP^A52G^ mutant induces neurodegeneration *in vivo*

**Figure 6:**
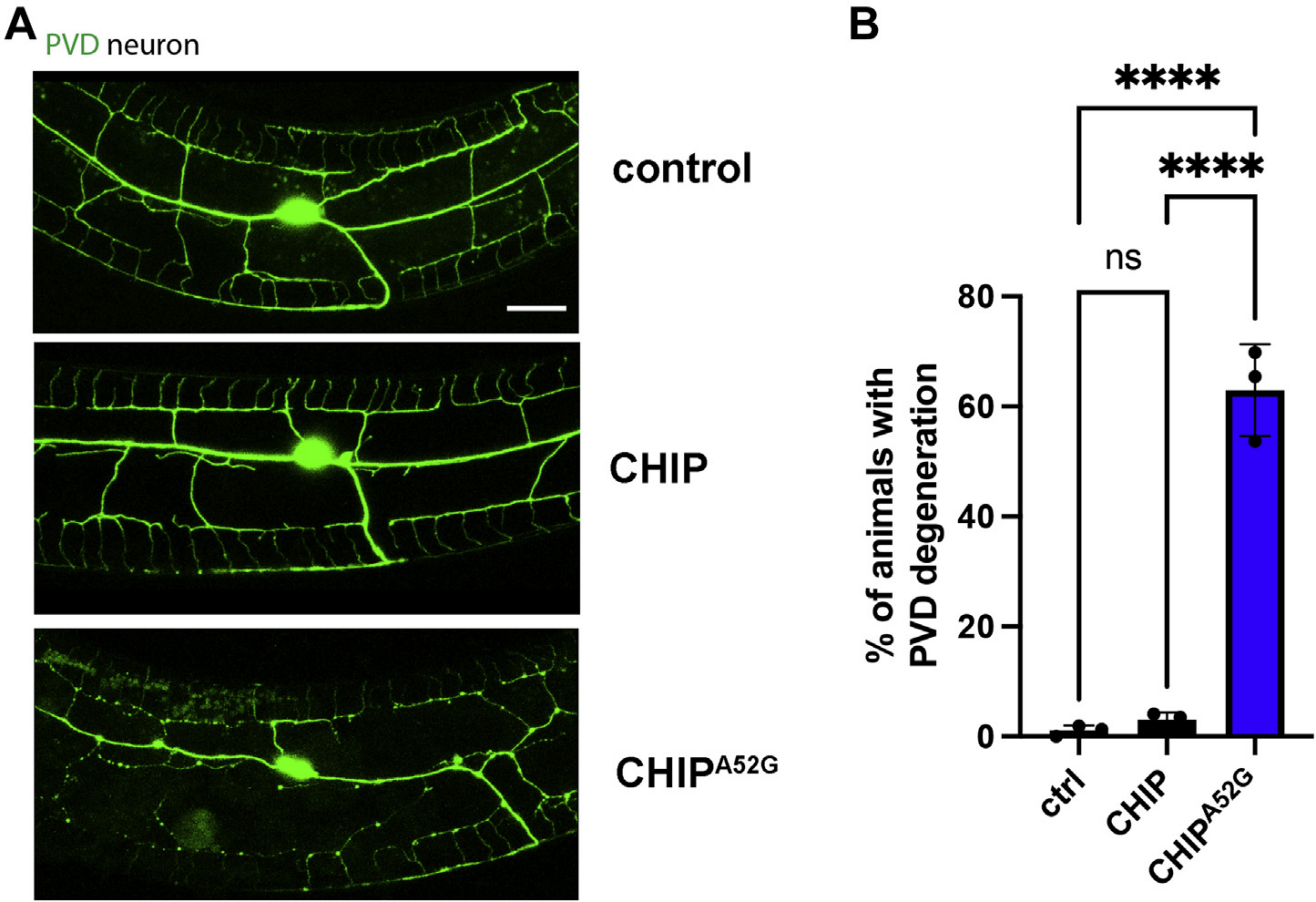
**Expression of CHIP**^**A52G**^**induces neurodegeneration in transgenic *C. elegans*.***A*, CHIP^A52G^ induces neurodegeneration in *C. elegans*. Confocal images show that the expression of the CHIP^A52G^ mutant causes PVD neurodegeneration in young adults (day 1 animals; scale bar = 10 μm). *B*, quantification of A. CHIP, C terminus of Hsc70 interacting protein, ∗∗∗∗*p* < 0.0001.

Our data demonstrate that CHIP^A52G^ has defects both *in vitro* and in cells; however, the effect of CHIP^A52G^ on causing neurodegeneration is unknown. To determine if CHIP^A52G^ expression results in neurodegeneration, we next produced transgenic *Caenorhabditis elegans* pan neuronally expressing either CHIP or CHIP^A52G^. Importantly, our transgenic *C. elegans* were created in the presence of endogenous CHIP to more accurately model this dominant form of SCA. We next imaged *C. elegans* PVD neurons to determine if the CHIP^A52G^ mutation resulted in neurodegeneration. Consistent with the CHIP^A52G^ mutation inducing neurodegeneration, we observed bead-like structures along neuronal processes that are enriched with autophagosomes and fragmented microtubules (42, 43), a phenotype that is reminiscent of neurodegeneration in mammalian model systems (Fig. 6*A*) (44, 45, 46, 47). Importantly, we did not observe this phenotype with animals expressing WT CHIP, consistent with the CHIP^A52G^ mutation being sufficient to induce neurodegeneration (Fig. 6, *A* and *B*). Together, these data indicate that the CHIP^A52G^ mutation causes neurodegeneration.

## Discussion

Here we have identified a *de novo* mutation in the *STUB1* gene that causes ataxia (Fig. 1). Our data indicate that the CHIP^A52G^ mutant has decreased helical content and is slightly destabilized in cells; however, it does not have a deficiency in thermal stability or oligomerization (Fig. 2). We further demonstrate that the A52G mutation disrupts the interaction with chaperones both *in vitro* and in cells (Fig. 3). We also found that the CHIP^A52G^ mutant retains ubiquitin ligase activity; however, its decreased affinity for chaperones disrupts substrate recruitment (Fig. 4). In human cells, we observed that CHIP^A52G^ exhibited a dominant phenotype where it caused an increased sensitivity to some cellular stressors (Fig. 5). Finally, we generated the first animal model of SCA48 and observed mutation-specific neurodegeneration in transgenic *C. elegans* (Fig. 6). Together, these data demonstrate that the A52G mutation causes SCA48 and provides insight into the molecular and cellular mechanisms that drive SCA48.

Our analysis reveals that in cells, the CHIP^A52G^ mutation causes either a toxic gain of function or a dominant negative phenotype, resulting in increased sensitivity to certain cellular stressors. This was somewhat surprising as we found that chemicals that act on similar pathways did not necessarily result in similar toxicity; however, previous work has demonstrated that this can occur (48, 49). We also found this result surprising because biochemically and in cells, the CHIP^A52G^ causes a decrease in chaperone binding, and the major function of CHIP is thought to be ubiquitinating chaperone-bound client proteins. This raises the question of how CHIP^A52G^ causes a gain of function or acts as a dominant negative.

Interestingly, previous work has identified heat stress as a condition that causes a decrease in the interaction between CHIP and chaperones (50). Studies have also found that during heat stress, CHIP localizes to cell membranes and to the nucleus (28, 50). One possibility is that the CHIP^A52G^ mutant behaves in a similar manner with a decreased interaction with chaperones and altered localization. In the future, it will be important to identify alterations in CHIP’s localization and interacting partners in SCA48 mutants to provide insight into alterations in cellular processes that drive neurodegeneration in SCA48.

Interestingly, the CHIP^A52G^ mutation resides in the same interface as every other identified SCA48 TPR mutant located between the first and second alpha-helical pairs in the CHIP TPR domain. SCA48 mutations occur in the buried residues that stabilize the TPR domain and form the groove for binding the -EEVD motif of molecular chaperones (Fig. 3, *B* and *C*). These observations suggests that other TPR mutations that cause SCA48 likely function through a similar mechanism. This is distinct from mutations in the TPR domain of CHIP that cause SCAR16. SCAR16 mutations in the TPR domain of CHIP are found on the surface or located in the interface of the helical bundles that comprise the second and third TPR repeat (37). One SCAR16 mutation, the N65S mutation, does fall next to the cluster of SCA48 TPR mutants; however, this residue is surface exposed and directly binds to chaperones. Together this raises the question of what effect destabilization of the interaction between the first and second helical bundles in CHIP’s TPR domain plays in modulating CHIP function.

In addition to SCA48, mutations in CHIP also cause SCAR16, a recessive form of ataxia. Previously, work from our group demonstrated that mutations in CHIP that cause SCAR16 do so by partially destabilizing CHIP resulting in decreased ubiquitin ligase activity *in vitro* and decreased stability in cells (37). We further demonstrated that reducing the temperature below the T_M_ of CHIP *in vitro* could partially restore CHIP chaperone binding and substrate ubiquitination (37). Unlike SCAR16 mutations, the A52G mutation did not result in any aberrant oligomerization or decrease in thermal stability; however, we did observe either a toxic gain of function or a dominant negative effect upon expressing CHIP^A52G^ in cells. This is important as autosomal dominant diseases like SCA48 could also be caused by a loss of function. In the future it will be important to determine if other SCA48 mutants also function by similar mechanisms. It will also be important to determine what cellular functions are altered by SCA48 mutations that cause toxicity.

Clinically SCAR16 and SCA48 present as a disease spectrum with cerebellar features including ataxia being the unifying symptom. In addition to ataxia, other more common symptoms include cognitive impairment, hyperkinesia, and isolated hyperreflexia. In SCAR16, biochemical and biophysical defects in specific domains of CHIP correlate with increased tendon reflex and cognitive dysfunction (51). This suggests that similar to SCAR16, the domain-specific defects in CHIP function may correlate with clinical outcomes in a SCA48 mutation-specific manner. While our study only investigates a single mutation that causes SCA48, in the future, additional analysis on other SCA48 mutations may provide insight into how domain specific mutations in CHIP correlate with SCA48 clinical outcomes.

## Experimental procedures

### Constructs

CHIP was cloned into pGEX6p-1, and Hsp70 was cloned into pMCSG7 as previously described (52). UbcH5 (plasmid 12643), Ube1 (plasmid 34965), and ubiquitin (plasmid 12647) were obtained from Addgene (53, 54). For mammalian expression CHIP was cloned into pcDNA3.1/Myc-His as previously described (52). Mutations were introduced using QuikChange Lightning Mutagenesis kit (Stratagene). For NanoBiT (Promega) assays, Hsp70 and CHIP were cloned into pBiT1.1-C [TK/LgBiT] and pBiT2.1-C [TK/SmBiT] using Nhe1 and Xho1 and additionally cloned into pBiT1.1-N [TK/LgBiT] and pBiT2.1-N [TK/SmBit] using Xho1 and Xba1. SmBiT-PRKACA and LgBit-PRKAR2A control vectors were used as a positive control, and LgBit-PRKAR2A and Halo-Tag-SmBit control vectors were used as a negative control.

### Mass photometry

Mass photometry experiments were carried out on a OneMP Mass Photometer (Refyn) using an Accurion i4 vibration isolation system to damp external vibration. The experiments were prepared by washing High Precision microscope cover glasses (Thorlabs) by iterative 10 min sonication baths with 50:50 Isopropanol:DI water then 100% DI water with DI water rinses between sonication. Immediately before preparing wells the coverslips were dried with low flow filtered air. Wells were then prepared by adhering CultureWellTM gaskets (Grace Bio-Labs) gaskets to the coverslip. A drop of Olympus IMMOIL-F30CC immersion oil was placed on the lens, and the coverslip was situated on the stage with the lens centered near the edge of a well. AquireMP software was used to focus the mass photometer by drop-dilution method using 15 μl buffer containing 20 mM Hepes pH 8, 200 mM NaCl, and 1 mM DTT. Once focused, 5 μl of prediluted 120 nM CHIP sample was added to the well to a final volume of 20 μl and 30 nM concentration. A 60 s video was then recorded in AquireMP at 100 frames per second. The video was analyzed in DiscoverMP with contrast values fit to a standard curve generated from BSA (Sigma P0834) and ApoFerritin (Sigma A3660) to calculate the mass of detected particles. The resultant fitted events were analyzed in Python utilizing SciPy and Pandas libraries to generate KDEs and gaussian fits which were then plotted in GraphPad Prism.

### Circular dichroism

CHIP and CHIP^A52G^ samples were diluted to approximately 10 μM in 10 mM sodium phosphate, pH 7.5. The concentration of each sample was measured *via* absorbance at 280 nm using a Take3 microplate in a BioTek Synergy H1 microplate reader. Diluted samples were loaded into a 1 mm Hellma absorption quartz cuvette and placed into an AVIV model 435 CD spectrometer. Full wavelength spectra were acquired at 25 °C with data collected in 1 nm increments from 190 nm to 260 nm. Full wavelength scan in millidegrees were collected in triplicate, averaged, converted to molar ellipticity, and plotted *versus* wavelength using GraphPad Prism. Thermal denaturation measurements were carried out by monitoring millidegree signal at 222 nm while ramping the temperature in 1 °C increments from 4 °C to 90 °C with a 30 s equilibration at each temperature prior to collecting data. Data were converted to molar ellipticity and plotted *versus* temperature using GraphPad Prism. Melting points were determined by nonlinear least squares regression using a sigmoidal Boltzmann fit within GraphPad Prism for data regions of 4 °C to 90 °C excluding 44 °C to 67 °C for the WT CHIP first transition, 44 °C to 90 °C for the WT CHIP second transition, 4 °C to 90 °C excluding 42 °C to 67 °C for the A52G mutant CHIP first transition, and 42 °C to 90 °C for the A52G mutant CHIP second transition.

### Western blotting

Samples for western blotting were collected from HEK293 cells by direct lysis with Laemmli buffer followed by sonication and boiling at 100 °C for 4 min. *In vitro* reactions were stopped by the addition of Laemmli buffer and boiling at 100 °C for 4 min. Both cellular lysates and *in vitro* reactions were next resolved by SDS-PAGE and transferred *via* Western blot to PVDF. Proteins were imaged by probing with the indicated antibodies.

### Cycloheximide chase assay

Cos-7 cells were maintained in Dulbecco’s modified Eagle’s medium (DMEM) (Corning) supplemented with 10% FBS (Millipore Sigma) in a 37 °C incubator with 5% CO_2_. Transfections were performed using X-tremeGene 9 (Roche) according to the manufacturer’s instructions. Twenty-four hours posttransfection, cells were treated with 50 μg/ml cycloheximide for 0, 1, 3, and 5 h. Cell lysates were collected, and Western blot analysis was performed.

### Sequence alignment

The Clustal Omega web server was utilized to align of the region of CHIP surrounding A52. UniProt was utilized to obtain the amino acid sequences for *Homo sapiens* (Q9UNE7), *Rattus norvegicus* (P29975), *Mus musculus* (Q9WUD1), *Danio rerio* (Q7ZTZ6), and *Drosophila melanogaster* (Q9XYW6) CHIP amino acid sequences.

### Fluorescence polarization

FP assays were performed utilizing 20 nM rhodamine B-labeled Hsc70 peptide diluted in assay buffer [50 mM Hepes, 75 mM NaCl, and 0.001% Triton X-100 (pH 7.4)]. Recombinant CHIP was added to peptide plated in 384-well, black, low-volume, flat bottom plates, and samples were covered and incubated in the dark for 30 min at 37 °C prior to reading on a Tecan Spark M20 plate reader with an excitation/emission setting of 544/612 nm. Results are show as the average with SD display. Experimental data was analyzed using GraphPad Prism.

### NanoBiT assays

Transfections were performed using Lipofectamine 3000 (Invitrogen) per manufacturer instructions. Twenty-four hours after transfection, cells were collected and resuspended in OptiMEM. Cells (100 ul) were plated in a 96-well white tissue culture–treated polystyrene plate and allowed to settle for 45 min in the incubator. Nano-Glo Live Cell Reagent (Promega) was added per manufacturer instructions, and samples were then placed on an orbital shaker for 15 s. Luminescence was then measured using a Tecan Spark M20 Plate reader with enhanced luminescence.

### Substrate ubiquitination assay

For ubiquitination of CHIP substrate, assays were performed as previously described (37, 55). For CHIP-dependent ubiquitination assays, we used 1 μM CHIP, 1 μM UbcH5, 1 μM Hsp70, and E1^mix^. E1^mix^ consists of 100 nM E1, 250 μM Ub, 2.5 mM ATP, and 2.5 mM MgCl2. All reactions were performed in a kinase buffer (50 mM Tris, 50 mM KCl, 0.2 mM DTT, pH 7.5) at 37 °C. The reactions were stopped by the addition of Laemmli buffer and boiling, followed by separation of proteins by SDS-PAGE and visualization by immunoblotting with the indicated antibodies.

### Lysine discharge and autoubiquitination

Pulse-chase assays were performed largely as previously described (56). To generate a thioester-linked UbcH5 ∼ Ub intermediate, the reaction was set up containing 0.1 μM E1, 10 μM UbcH5, and 5 mM MgATP in the reaction buffer containing 20 mM Hepes pH 8, 200 mM NaCl. 1 μM E1 was used for the lysine discharge assays. The reaction was started by the addition of 10 μM of fluorescein-tagged Ub and incubated at room temperature for 10 min, then quenched with 50 mM EDTA. It was then cooled on ice for 5 min and used for subsequent chase reaction. After the quench, the pulse reaction mixture was added to the chase solution at a 1:6 ratio on ice. The final reaction concentrations were 1.7 μM UbcH5 ∼ Ub and 5 μM CHIP. For the lysine discharge assay, 5 mM lysine was added to the chase reaction. Aliquots of the chase reactions were quenched by adding them to SDS loading buffer at specified time points. The SDS samples were separated by SDS-PAGE, and the gels were scanned on an Amersham Typhoon imager (GE Healthcare). Bands corresponding to UbcH5 ∼ ∗Ub were quantitated. Values were plotted as a fraction of quantitated Ubch5 ∼ ∗Ub band at 60 min for autoubiquitination assays and 30 min for lysine discharge assays.

### Cellular stress assays

The assays were performed as previously described with minor modifications (41). HEK293 cells were transfected with either empty vector, pcDNA3.1-CHIP, or pcDNA3.1-CHIP^A52G^. Two days after transfection, 10,000 cells were seeded into 96-well tissue culture treated microtiter plates (GenClone, Cat. #25-109) in 100 μl of DMEM (Gibco, Cat. #11995-065) [supplemented with 10% fetal bovine serum (HyClone, Cat. #SH30396.02HI)], Pen Strep (Gibco, Cat. #15140-122), and non-essential amino acids (Gibco, Cat. #11140050)] and were incubated overnight. For the glucose tolerance assays cells in each well were washed with PBS and cultured in medium supplemented with the indicated concentrations of glucose (Sigma-Aldrich, Cat. #G8270) for 6 h. For the rest of stressors [tunicamycin (EMD Millipore Corp, Cat. #654380), methyl methanesulfonate (Alfa Aesar, Cat. #h55120.06), thapsigargin (EMD Millipore Corp, Cat. #586005), hydrogen peroxide (Macron Fine Chemicals, Cat. #5240), cadmium chloride (Sigma-Aldrich, Cat. #202908) and ammonium chloride (Sigma-Aldrich, Cat. #A9434)], cells in each well were washed with PBS, and cultured by DMEM medium supplemented with 2% BSA (Sigma-Aldrich, Cat. #9048468) for 24 h. Then cells were then exposed to stressor for 6 h, washed with PBS and incubated with DMEM medium supplemented with 2% BSA again for 24 h. For all stressors cell viability was measured using CellTiter-Glo 2.0 (Promega, Cat. #G9242). At least 4 biological replicates were used for viability tests in the presence of each stressor.

### C. elegans strains and maintenance

*C. elegans* were maintained on nematode growth media with *E. coli* OP50 at 20 °C as previously described (57) unless otherwise indicated. Transgenic strains were generated by microinjection. All plasmids used for microinjection were made by Gateway cloning technology. DNA fragments of specific genes and related promoters were amplified using PCR, then inserted in pCR8 entry vector or destination vector, respectively. P*unc-122*::RFP were used as the co-injection marker.

### Quantification of PVD neurodegeneration

*wdIs51[*F49H12.4::GFP *+ unc-119(+)]* transgene marker was used for observation of PVD neurodegeneration. The PVD neurodegeneration were quantified using 63× magnification on the ZESS microscope as previously described (42, 58). Degenerated PVD neurons will show bubble and bead-like structures on the PVD dendrite. For each independent experiment, at least 50 animals were used for quantification of neuron degeneration. Three to four replicates were conducted for each experiment.

### Fluorescence microscopy

We scored fluorescent reporters in live animals using a Zeiss Axio Imager 2 microscope equipped with Chroma HQ filters. Confocal images were collected of animals immobilized by 1 % 1-phenoxy-2-propanol (TCI America) in M9 buffer using a Zeiss LSM700 confocal microscope. Pictures shown in the figures are projections of z-stack images (1 μm/section). Data are presented as the means ± SD. *t* test was used for quantification. All figures were generated using GraphPad Prism 7 (GraphPad Software), BioEdit, and Adobe Illustrator.

## Data availability

All data are contained within the article.

## Supporting information

This article contains supporting information.

## Conflict of interest

The authors declare that they have no conflicts of interest with the contents of this article.

## Supporting information

Supplemental Figure 1 **Optimization of CHIP/Hsp70 NanoBiT constructs**. HEK293 cells were transfected with all possible combinations of CHIP and Hsp70 fused to the LgBiT and SmBiT tags and with positive and negative controls (Promega). Tagging Hsp70 with an N-terminal LgBiT and tagging CHIP with an N-terminal SmBiT provided the optimal signal. CHIP, C terminus of Hsc70 interacting protein
