# Supplementary figures and images for: The molecular basis of spinocerebellar ataxia type 48 caused by a de novo mutation in the ubiquitin ligase CHIP
Source: J Biol Chem. 2022 Apr 7;298(5):101899. doi: 10.1016/j.jbc.2022.101899 (PMC9097460; doi:10.1016/j.jbc.2022.101899)

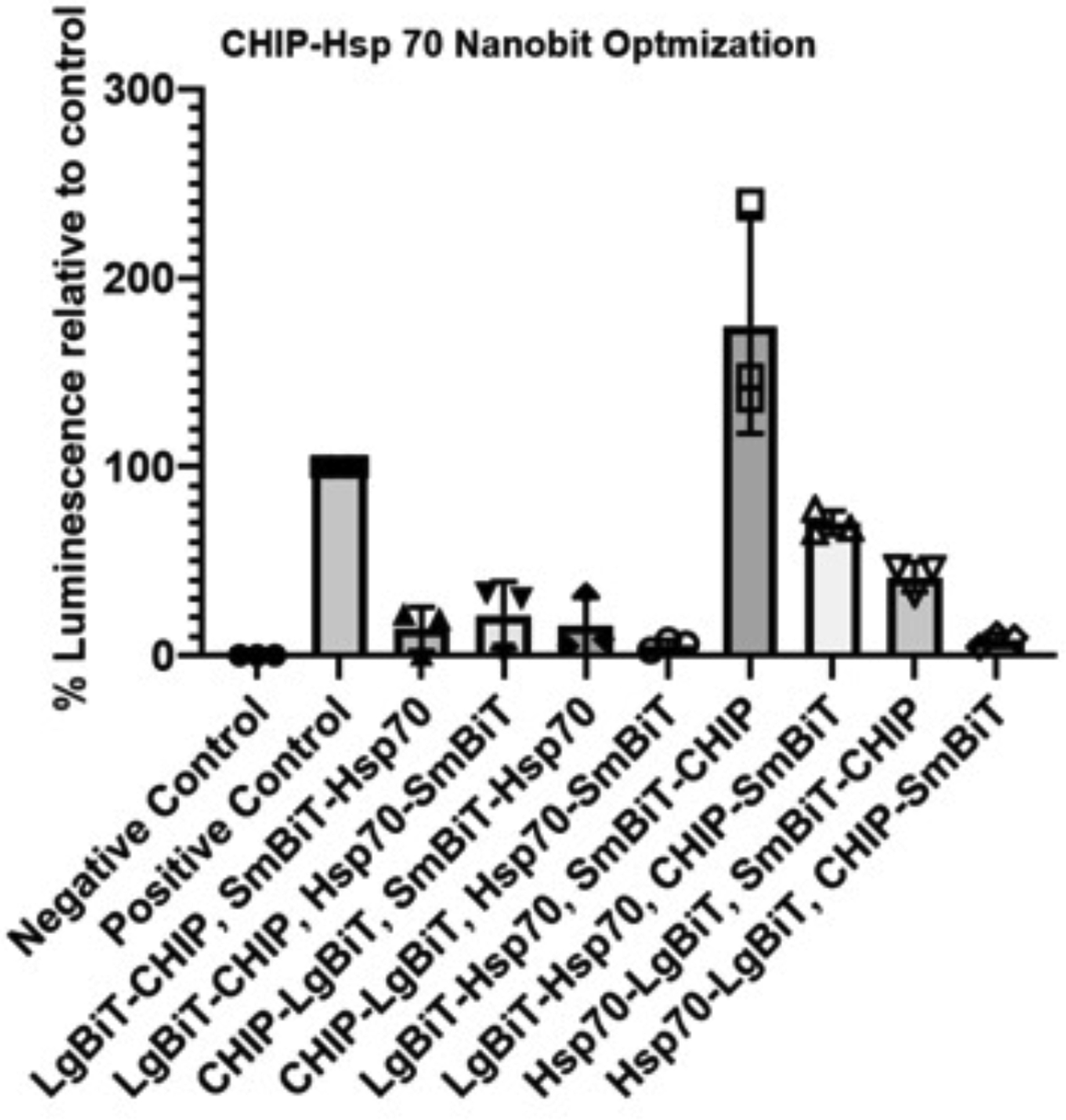

Supplement: Supplemental Figure 1 — Optimization of CHIP/Hsp70 NanoBiT constructs. HEK293 cells were transfected with all possible combinations of CHIP and Hsp70 fused to the LgBiT and SmBiT tags and with positive and negative controls (Promega). Tagging Hsp70 with an N-terminal LgBiT and tagging CHIP with an N-terminal SmBiT provided the optimal signal. CHIP, C terminus of Hsc70 interacting protein [file figs1.jpg]
